# Neuropeptide S- and Neuropeptide S receptor-expressing neuron populations in the human pons

**DOI:** 10.3389/fnana.2015.00126

**Published:** 2015-09-25

**Authors:** Csaba Adori, Swapnali Barde, Nenad Bogdanovic, Mathias Uhlén, Rainer R. Reinscheid, Gabor G. Kovacs, Tomas Hökfelt

**Affiliations:** ^1^Department of Neuroscience, Karolinska InstitutetStockholm, Sweden; ^2^Geriatric Department, Institute for Clinical Medicine, Oslo UniversityOslo, Norway; ^3^Science for Life Laboratory, Department of Neuroscience, Karolinska InstitutetStockholm, Sweden; ^4^Science for Life Laboratory, Albanova University Center, Royal Institute of TechnologyStockholm, Sweden; ^5^Department of Pharmaceutical Sciences, University of California, IrvineIrvine, CA, USA; ^6^Department of Pharmacology, University of California, IrvineIrvine, CA, USA; ^7^Department of Molecular Biology and Biochemistry, University of California, IrvineIrvine, CA, USA; ^8^Institute of Neurology, Medical University of ViennaVienna, Austria

**Keywords:** Neuropeptide S (NPS), anxiety, arousal, deep brain stimulation (DBS), human brain, parabrachial, periaqueductal gray, sudden infant death syndrome

## Abstract

Neuropeptide S (NPS) is a regulatory peptide with potent pharmacological effects. In rodents, NPS is expressed in a few pontine cell clusters. Its receptor (NPSR1) is, however, widely distributed in the brain. The anxiolytic and arousal-promoting effects of NPS make the NPS–NPSR1 system an interesting potential drug target in mood-related disorders. However, so far possible disease-related mechanisms involving NPS have only been studied in rodents. To validate the relevance of these animal studies for i.a. drug development, we have explored the distribution of NPS-expressing neurons in the human pons using *in situ* hybridization and stereological methods and we compared the distribution of NPS mRNA expressing neurons in the human and rat brain. The calculation revealed a total number of 22,317 ± 2411 NPS mRNA-positive neurons in human, bilaterally. The majority of cells (84%) were located in the parabrachial area in human: in the extension of the medial and lateral parabrachial nuclei, in the Kölliker-Fuse nucleus and around the adjacent lateral lemniscus. In human, in sharp contrast to the rodents, only very few NPS-positive cells (5%) were found close to the locus coeruleus. In addition, we identified a smaller cell cluster (11% of all NPS cells) in the pontine central gray matter both in human and rat, which has not been described previously even in rodents. We also examined the distribution of NPSR1 mRNA-expressing neurons in the human pons. These cells were mainly located in the rostral laterodorsal tegmental nucleus, the cuneiform nucleus, the microcellular tegmental nucleus region and in the periaqueductal gray. Our results show that both NPS and NPSR1 in the human pons are preferentially localized in regions of importance for integration of visceral autonomic information and emotional behavior. The reported interspecies differences must, however, be considered when looking for targets for new pharmacotherapeutical interventions.

## Introduction

Neuropeptide S (NPS) is a 20 amino acid modulatory neuropeptide that was identified in 2004 by the orphan receptor strategy and named after its conserved N-terminus **s**erine residue (Xu et al., 2004). NPS is found in all vertebrates except fish (Reinscheid, 2007).

NPS has potent pharmacological effects. Thus, central administration of NPS in rodents induces hyperlocomotion and promotes arousal, inhibits food intake (Smith et al., 2006; Peng et al., 2010) and produces anxiolytic- and panicolytic-like as well as reward-like effects (Cao et al., 2011; Pulga et al., 2012; Wegener et al., 2012). Moreover, NPS mediates control of fear expression and extinction in the amygdala (Jüngling et al., 2008) and may have an important role in learning and memory processes (Okamura et al., 2011; Lukas and Neumann, 2012). The unique behavioral response of animals after intracerebroventricular (i.c.v.) NPS administration, namely the simultaneous anxiolytic and arousal-promoting effects, makes the NPS–NPSR1 system an interesting potential drug target in mood-related disorders and has initiated research on the role of NPS in appropriate rodent disease models (Reinscheid and Xu, 2005a,b; Donner et al., 2010; Domschke et al., 2011).

Despite its powerful pharmacological effects, NPS is one of the least abundant neuropeptides in the rodent brain with regard to levels and number of expressing neurons (Liu et al., 2011). Previous studies in rat and mouse (Clark et al., 2011; Liu et al., 2011) showed that NPS is expressed by a few well-defined pontine neuronal clusters that project to several distinct rostral forebrain areas, including the septum, hypothalamus and thalamus. Its G-protein-coupled receptor, NPSR1 (Reinscheid and Xu, 2005a), is however widely distributed throughout the brain (Xu et al., 2007; Clark et al., 2011) and mediates predominantly excitatory signals (Reinscheid and Xu, 2005a). In agreement, the NPS neurons are glutamatergic (Liu et al., 2011).

For a long time, rodents have been used to explore normal brain functions and mechanisms underlying brain diseases, including mood-related disorders, such as depression-like behavior and anxiety. However, the chemical neuroanatomy of the diurnal human may differ from the nocturnal rodents (Novak et al., 1999; Alam et al., 2011). This is especially true for neurons expressing certain neuropeptides and/or their receptors. For instance, galanin is expressed in dorsal raphe (DR) neurons in rat but not in human, and galanin receptor 3 (GalR3) is highly expressed in the human locus coeruleus (LC; Le Maitre et al., 2013) vs. GalR1 and -2 in rat (O'Donnell et al., 1999). Also, substance P co-exists with serotonin neurons in the DR of human but not of rat (Sergeyev et al., 1999). Moreover, nNOS is highly expressed in the DR of rat but not in human. In contrast, nNOS is expressed in the human but not rat LC (Le Maitre et al., 2013). Taken together, the neuroanatomical and neurochemical analysis of the human brain is essential for identifying targets for drug development and for understanding mechanisms underlying disease.

In our present study we generated riboprobes against the human NPS and NPSR1 and performed sensitive radioactive *in situ* hybridization to explore the distribution of neurons that express the mRNA of NPS and its receptor in the human pons. Our results show that the distribution of NPS and NPSR1 mRNA-expressing neurons is just partially in agreement between human and rodents and thus that some distinct interspecies differences exist.

## Materials and methods

### Production of riboprobes

A 183-bp fragment of the human NPS precursor cDNA (gene ID: 594857, corresponding to nucleotides 133–315) and a 300-bp fragment for the human NPS receptor cDNA (gene ID: 387129, corresponding to nucleotides 763–1063) were generated by PCR from human total brain cDNA and subcloned into PCR1II-TOPO vector (Invitrogen, Carlsbad, CA) in our lab. The sequence of the probes was confirmed by restriction digestion and sequencing (KIGene, Stockholm, Sweden). Linearized antisense and sense riboprobes were generated using T7 and Sp6 RNA polymerase, respectively.

Riboprobes against rat NPS was generated as described before (Xu et al., 2007). Briefly, a 184-bp BamHI-NotI fragment of the rat NPS precursor cDNA (corresponding to nucleotides 92–276) was generated by PCR and subcloned into pBluescript SK (Stratagene, La Jolla, CA). Antisense or sense riboprobes were generated by using T7 or T3 RNA polymerase, respectively.

### Sample collection and sectioning for *in situ* hybridization

Brains from three subjects without significant neurological symptoms or substantial neuropathological alterations in the rostral and caudal brainstem were collected (Clinical and neuropathological data are summarized in Table 1). All procedures involving human tissues were in accordance with the 1964 Helsinki declaration and its later amendments or comparable ethical standards (ethical permissions: Regionala etikprövningsnämnden i Stockholm, permission number: 2013/474-31/2, Stockholm, Sweden; Ethik-Kommission der Medizinischen Universität Wien, permission number: 396/2011, Vienna, Austria).

**Table 1:** **Clinical-neuropathological data of the applied subjects/brains**.

	**Age**	**Gender**	**Cause of death**	**Clinical neurological symptoms**	**Neuropathology**	**Post mortem delay (PMD)**	**Brain pH**
Case 1	66	Male	Myocardial infarction	No	Histological signs of mild brain edema	6.5 h	6.20
Case 2	76	Female	Cardiac failure	No	Mild astrocytic and neuronal tau pathology (CBD-like) in the frontal and temporal cortex, basal ganglia and substantia nigra	12 h	6.25
Case 3	85	Male	Myocardial infarction	Mild cognitive impairment	Neurofibrillary degeneration in the hippocampus and entorhinal cortex (Stage II Braak) without Abeta plaques (PART)	8 h	6.15

Whole brainstems were dissected and rapidly frozen in isopentane pre-cooled in liquid nitrogen, with much care of sterility and avoiding distortion or any destruction of the tissue. The brainstem was separated rostrally at the level of the substantia nigra and nucleus ruber and caudally at the level of the pontomedullary transition. The remaining brainstem had, in addition to several neocortical areas, hippocampus, thalamus, basal ganglia, and cerebellum, been examined for neuropathological alterations using standard neuropathological methods (Kovacs, 2014). The pH of all the three examined brains was determined on blocks of frozen tissue of the cerebellum after thawing as described (Monoranu et al., 2009). As postulated by Alafuzoff and Winblad (1993) and Ravid et al. (1992), pH is stable during storage and the values were therefore comparable. The measured pH in the three cases did not show significant variation (6.20, 6.25, and 6.15, cases 1–3, respectively). The frozen brainstems were cut systematically on a cryostat (Microm, Heidelberg, Germany) extending from the rostral part of the inferior olivary nucleus to the rostral part of the substantia nigra (approximately between Obex +15 and Obex +37 mm; seven parallel 20-μm-thick sections in each 500 μm).

Adult male Wistar rats were decapitated, and their brains were quickly frozen on dry ice. A series of 20-μm-sections were prepared on a cryostat (as above) between Bregma −10.00 and −8.00 mm. All animal experiments in the present study were performed according to the European Communities Council Directive of 24 November 1986 (86/609/EEC), and the experiments were approved by the National Scientific Ethical Committees on Animal Experimentation (Stockholms norra djurförsöksetiska nämnd, Sweden, permit number: N171-172/11).

### *In situ* hybridization procedure in human and rat tissue

Human and rat brainstem sections were fixed for 10 min in cold 4% (wt/vol) paraformaldehyde in 0.1 M PBS, pH = 7.4, for 5 min in 1x PBS; followed by 5 min in diethyl pyrocarbonate-treated water; 5 min in 0.1 M HCl; 2 × 3 min in 1x PBS; 20 min in 0.25% acetic anhydride in 0.1 M triethanolamine; and 2 × 3 min in 1x PBS. Then, sections were dehydrated in 70–80–99.5% ethanol, each for 2 min. Sections were air dried and then were incubated in prehybridization cocktail [50% (vol/vol) deionized formamide (pH 5.0), 50 mM Tris· HCl (pH 7.6), 25 mM EDTA (pH 8.0), 20 mM NaCl, 0.25 mg/mL yeast tRNA, and 2.5x Denhardt's solution] for 4–6 h at 55°C followed by hybridization in a humidified chamber overnight (14–16 h) at 55°C. Radiolabeled probes were prepared by *in vitro* transcription using the MAXIScript Sp6/T7 kit (Applied Biosystems, Carlsbad, CA) and [α^35^ S]UTP (NEG039H, Perkin Elmer, Waltham, MA). The labeled probes were separated from unincorporated nucleotides using NucAway spin columns (Ambion, Carlsbad, CA). The labeled probes were diluted to a final concentration of 1.0 × 106 cpm/200 μL in a solution containing 50% (vol/vol) deionized formamide (pH 5.0), 0.3 M NaCl, 20 mM DTT, 0.5 mg/mL yeast tRNA, 0.1 mg/mL poly-A-RNA, 10% (vol/vol) dextran sulfate, and 1x Denhardt's solution (hybridization cocktail). After hybridization, sections were washed 2 × 30 min in 1x SSC at 55°C, 1 h in 50% (vol/vol) formamide/0.5x SSC at 55°C, 15 min in 1x SSC at 55°C, 1 h in “RNase A” buffer at 37°C, 2 × 15 min in 1x SSC at 55°C, and then dehydrated in a graded series of alcohol (2 min in each), and finally were air dried. The slides were first exposed against KODAK BioMax MR Film then dipped in KODAK NTB emulsion (Kodak, Rochester, NY; diluted 1:1 with water), exposed for 7 days, 8 weeks, or 12 weeks (rat NPS, human NPS, human NPSR1, respectively) and finally developed in Kodak D19 developer, fixed in Kodak Unifix, and mounted in glycerol-PB. A serial of hybridized sections and a serial of adjacent sections were stained for cresyl violet.

### Mapping of NPS and NPSR1 mRNA-expressing cells in the human brainstem

NPS antisense probe-labeled sections with or without cresyl violet counterstaining and parallel adjacent sections only stained with cresyl violet, collected at a distance of 500 μm between Obex +15 and Obex +37 mm, were examined using a Nikon Eclipse E600 microscope equipped with a Heim Fiberoptic darkfield apparatus/Nikon Universal Condensor C-CU lighfield apparatus and an ORCA-ER digital camera using Hamamatsu Photonics Wasabi 150 software. The detailed anatomical mapping was performed based on a human atlas (Paxinos et al., 2012).

### Estimation of the total number of NPS neurons in the human pons

NPS mRNA-expressing neurons were counted in NPS antisense probe-labeled and cresyl violet counterstained sections with 500 μm between each section. The labeled and examined sections started before the beginning of the NPS cell clusters and finished after the clusters. All sections with labeled cells for a particular cluster were included in the quantification (11–15, 11–14, and 20–22 sections, periventricular, pericoerulear and parabrachial (PB) cells, respectively). In a certain section all labeled neurons were counted. The total number of NPS mRNA-expressing neurons in a particular cluster was determined based on the principle of the *optical fractionator* (Gundersen, 1986; West, 1993) by the formula: *N* = Σ*Q*^−^^*^ *(1/ssf)*, where *N*, total cells; Σ*Q*^−^, counted cells; *ssf*, section sampling factor.

### Rats and intracerebroventricular colchichine injections

The experiments were performed on adult male Wistar rats (body weight 300–350 g) at Karolinska Institutet Stockholm, Sweden. Three rats received an i.c.v. injection of the mitosis inhibitor (and axoplasmic transport-blocker) colchicine (Dahlström, 1969) under Hypnorm/Midazolam anesthesia (contains Midazolam 6.25 mg/kg, fentanyl citrate 0.4 mg/kg, and fluanisone 12.5 mg/kg i.p). Colchicine was dissolved in 0.9% NaCl to a final concentration of 100 μg in 10 μl. The drug was slowly infused into the left cerebral ventricle using a Hamilton syringe with a 26G needle attached. Injection coordinates were: AP = 1.0 mm from bregma, *L* = 1.6 mm from midline, and *V* = 4.5 mm deep to the surface of the brain, according to published atlas for rat (Paxinos and Watson, 2007). The syringe was left in the brain for 5 min after injection to prevent back-flow of the colchicine. Twenty-four hours later, the rats were perfused and processed for immunohistochemistry. Before and after operations, the rats were maintained under standard conditions on a 12-h day/night cycle (lights on 07:00) with water and food available *ad libitum*.

### Immunohistochemistry and fluorescent microscopy

Colchichine-treated rats were deeply anesthetized using sodium pentobarbital (60 mg/kg i.p.; Apoteket, Stockholm, Sweden). They were perfused via the ascending aorta with 60 mL of Tyrode's buffer (37°C), followed by 60 mL of a mixture of 4% paraformaldehyde (PFA) and 0.2% picric acid diluted in 0.16 M phosphate buffer (PB; pH 6.9; 37°C, to keep the blood vessels diluted) and 300 mL of the same fixative at 4°C. The brains were dissected out and postfixed in the same fixative for 120 min at 4°C, and finally immersed in 10% sucrose diluted in phosphate-buffered saline (PBS; pH 7.4) containing 0.01% sodium azide (Sigma-Aldrich, St. Louis, MO) and 0.02% Bacitracin (Sigma-Aldrich; 4°C) for 48 h. The brains were snap-frozen with CO_2_, and sectioned at 20 μm in a cryostat (Microm, Heidelberg, Germany) between Bregma −10.00 and −8.00 mm. The sections were then mounted on SuperFrost Plus slides (VWR international, Leuven, Belgium).

For immunostaining, sections were washed in PBS and incubated overnight at room temperature with rabbit polyclonal antiserum against NPS (Abcam, cat no: ab18252) at the dilution of 1:10.000 in 0.01M PBS containing 0.3% TritonX-100, 0.02% bacitracin and 0.01% sodium-azide. To visualize the immunoreactivity, the sections were processed using a commercial kit (PerkinElmer Life Science, Boston, MA) based on tyramide signal amplification (TSA; Adams, 1992). Briefly, the sections were washed in TNT buffer (0.1 M Tris-HCl, pH 7.5; 0.15 M NaCl; 0.05% Tween 20) for 15 min, incubated with TNB buffer (0.1 M Tris-HCl, pH 7.5; 0.15 M NaCl; 0.5% Dupont Blocking Reagent, PerkinElmer) for 30 min at RT and incubated with an antirabbit IgG-HRP polymer conjugate (Invitrogen, Frederick, MD, USA) diluted 1:2 in TNB buffer for 30 min. The sections were washed in TNT buffer and incubated in a tyramide-fluorescein (FITC) conjugate (PerkinElmer) diluted 1:100 in amplification diluent for 15 min at RT. The NPS antiserum and its specificity were characterized extensively before (Clark et al., 2011). In agreement, no staining was detected in our sections when the primary antibody was pre-incubated overnight with 10^−6^ M or 10^−5^ M rat NPS peptide (Bachem, H6164; data not shown). After the immunoreactions, sections were coverslipped using 2.5% DABCO in glycerol (Sigma). The sections were examined using a Nikon Eclipse E600 fluorescence microscope with objective lenses 4x, 10x, 20x, and 63x (Nikon, Tokyo, Japan) equipped with appropriate filters and an ORCA-ER, C4742-80 digital camera (Hamamatsu Photonics K.K., System Division, Hamamatsu City, Japan), using Hamamatsu Photonics Wasabi 150 software. The distribution of NPS expressing neurons was determined based on a rat brain atlas (Paxinos and Watson, 2007).

## Results

### Distribution of NPS mRNA-expressing neurons in the human brainstem

**Figure 1:**
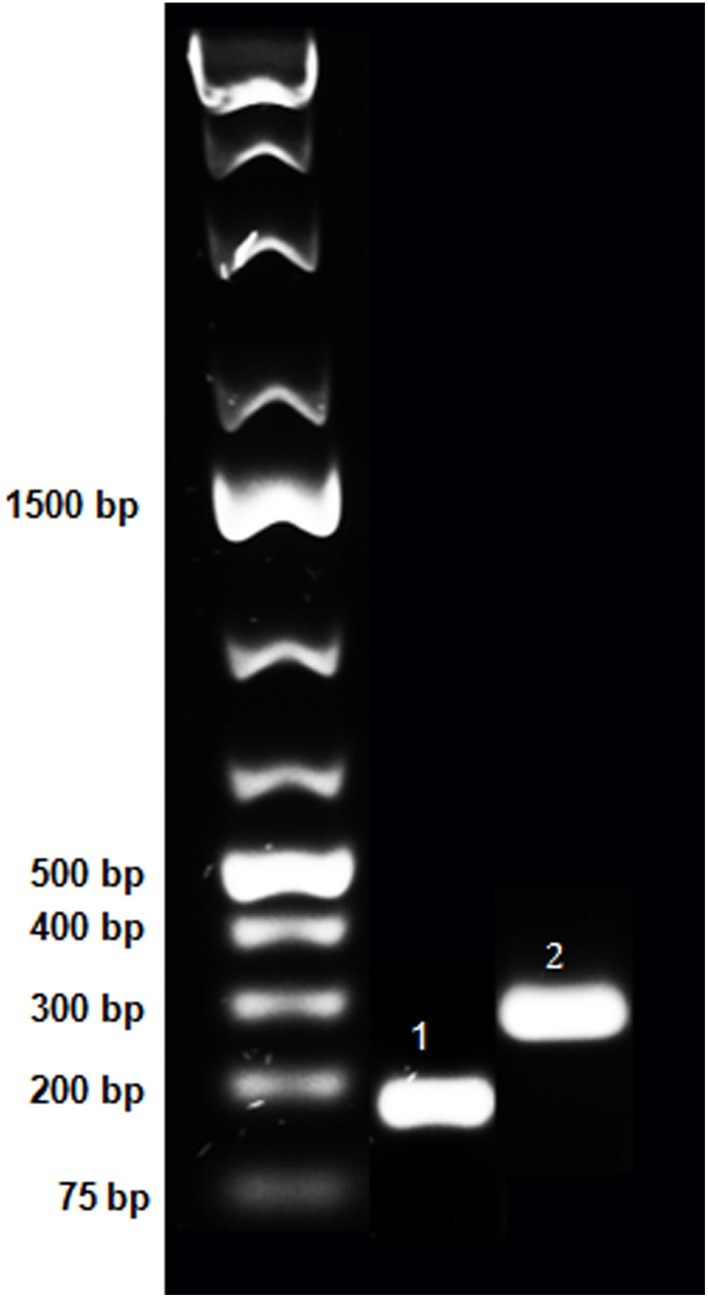
**Purification of PCR fragments of hNPS and hNPSR1 on agarose gel**. Purified PCR fragments of human NPS and NPSR1 were run on 1% agarose gel to confirm size and purity. These fragments were subcloned into TOPO-TA vector (Life Technologies) and, after verification of the sequence, RNA probes were generated. The lanes represent the O'GeneRuler 1 kb Plus DNA Ladder (Fermentas; left), the 183 bp PCR product of human NPS (middle, indicated with “1”) and the 300 bp fragment of human NPSR1 (right, indicated with “2”).

In order to determine the distribution of NPS mRNA-expressing neuronal populations in the human brainstem, we first produced riboprobes against human NPS (Figure 1). Then, we systematically cut three human brainstems approximately at the level of LC - DR which, based on previous rodent studies (Xu et al., 2007; Clark et al., 2011), is the expected localization of the NPS-expressing neurons.

**Figure 2:**
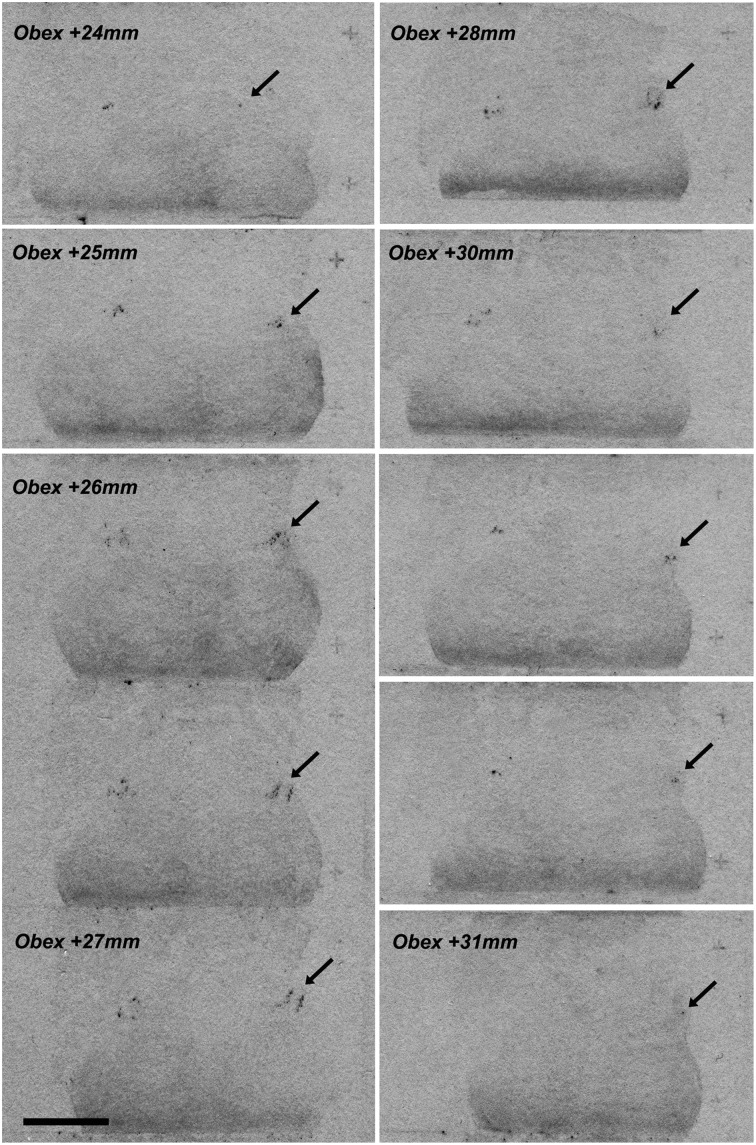
**NPS mRNA-positive neurons at different rostro-caudal levels of human pons (X-ray film)**. A series of coronal brainstem sections, from Obex +24 to Obex +31, hybridized with ^35^S-labeled hNPS riboprobes and exposed to KODAK X-ray film. A very limited but distinct signal is detected bilaterally in the proximity of the superior cerebellar peduncule/parabrachial region (arrows). Two subclusters of labeled cells are seen around Obex +27 mm. Scale bar: 10 mm.

A serial of coronal brainstem sections hybridized with ^35^S-labeled hNPS riboprobes was first exposed to a KODAK film for an approximate estimation of the distribution of the labeled cells. A strong signal with a limited extension was detected in the proximity of the superior cerebellar peduncle—PB region (Figure 2).

**Figure 3:**
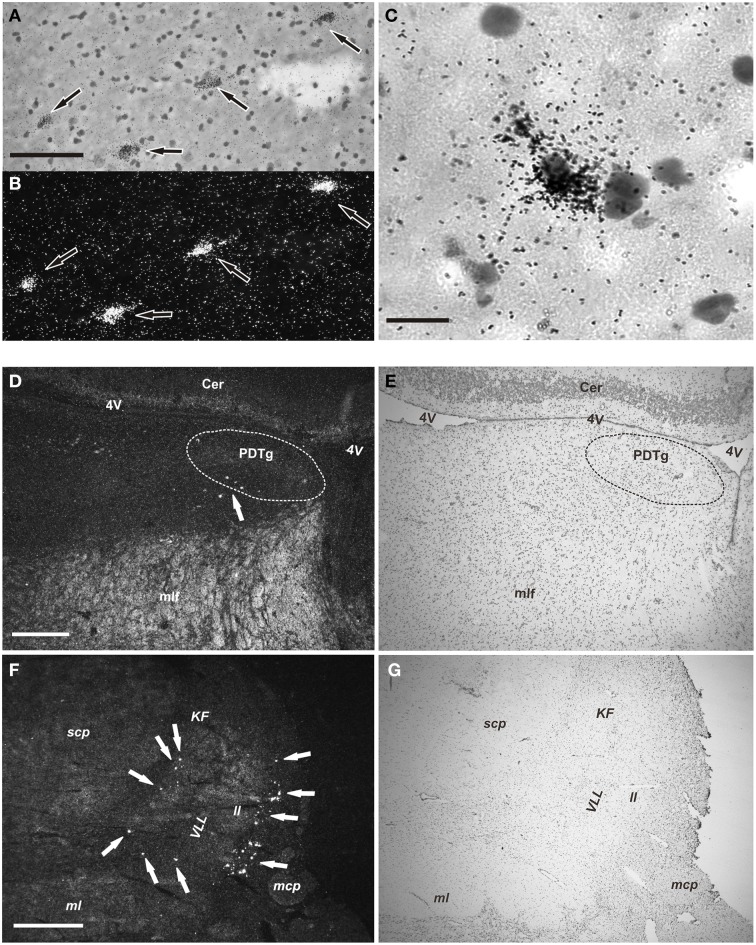
**NPS mRNA-positive neurons in human pons (emulsion-dipped)**. **(A,B)** Labeled neurons (arrows) are seen in the periventricular cell cluster in the same section (**A**, brightfield, cresyl violet; **B**, darkfield). **(C)** High magnification showing one NPS mRNA-positive neuron (cresyl violet, brightfield). **(D,E)** Low power overview of the central pontine gray matter showing a few cells (arrow) in the periventricular NPS cell cluster (**D**, darkfield); **(E)** shows the same section (cresyl violet, brightfield). The posterodorsal tegmantal nucleus (PDTg) is surrounded by dashed line in **(D,E). (F,G)** Low power overview of the parabrachial area showing many labeled neurons (arrows) of the parabrachial cell cluster (**F**, darkfield); **(G)** shows same section (cresyl violet; brightfield). Scale bars: 100 μm **(A)**, applies to **(A,B)**; 25 μm **(C)**; 500 μm **(D)**, applies to **(D,E)**; 1000 μm **(F)**, applies to **(F,G)**.

Subsequently, the distribution of NPS mRNA-expressing cells was determined in more detail on sections dipped in KODAK NTB emulsion. After an 8 week exposure, the radioactive NPS signal was strong, and positive cells were easily recognized both with darkfield and lightfield microscopy in section counterstained with cresyl violet (Figure 3). The distribution of NPS mRNA-expressing cells was the same in all the three examined brainstems.

**Figure 4:**
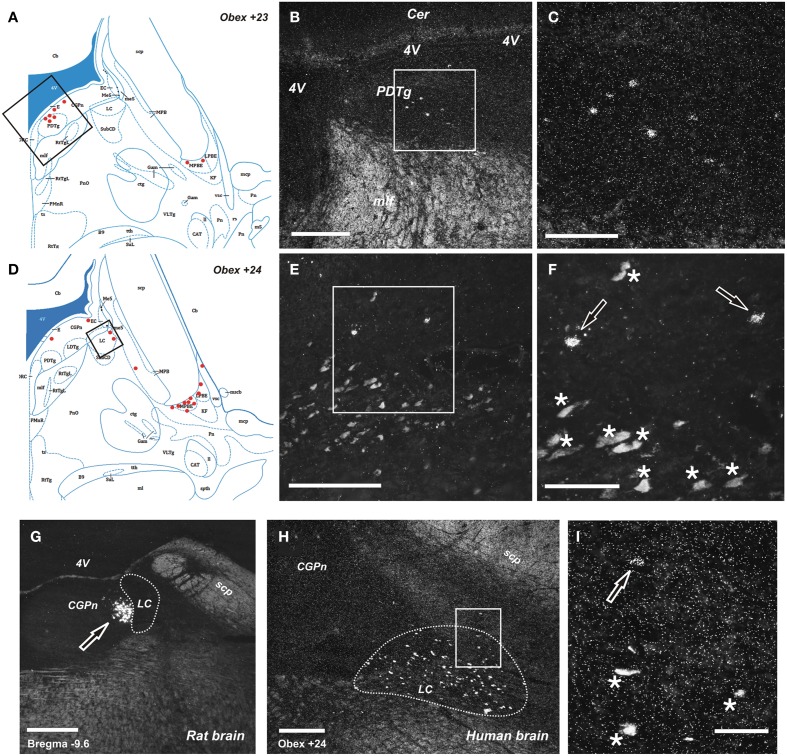
**Distribution of NPS mRNA-positive neurons in human and rat pons**. **(A,D)** Schematic drawings from the atlas by Paxinos et al. (2012), indicating distribution of NPS mRNA-positive cell bodies (red dots) at two different levels (Obex +23 and +24). All schematic drawings are presented in higher magnification in the Supplementary Material. Boxed area in **(A,D)** show **(B,E)**, respectively. **(B, C,E,F)** NPS mRNA-positive neurons belonging to the periventricular cluster in the central pontine gray matter adjacent to the posterodorsal tegmental nucleus **(B,C)**, and to the sparse pericoerulear cells dorsally, adjacent to the locus coeruleus (**E,F**; the numerous, pigmented and autofluorescent locus coeruleus neurons are indicated with asterisks and the NPS mRNA-positive neurons indicated with arrows, both in **F**). Boxed area in (**B,E**) show **(C,F)**, respectively. **(G**–**I**) Comparison of peri-coerulear NPS mRNA-postive neurons in the rat **(G)** and human **(H,I)**. The boxed area in **(H)** is shown in **(I)**. Note the compact cluster of neurons with high mRNA-expression level of NPS in rat **(G**, arrow), contrasting the very few scattered neurons around the locus coeruleus in human (**I**, arrow). The locus coeruleus is surrounded by dashed line in **(G,H)**; the human noradrenergic locus coeruleus neurons appear as autofluorescent pigmented cells, indicated with stars in **(I)**. **(A,D)** are reproduced from Paxinos et al. (2012), with permission. Scale bars: 200 μm **(C)**, **(F–H)**; 500 μm **(B,E)**; 50 μm **(I)**.

**Figure 5:**
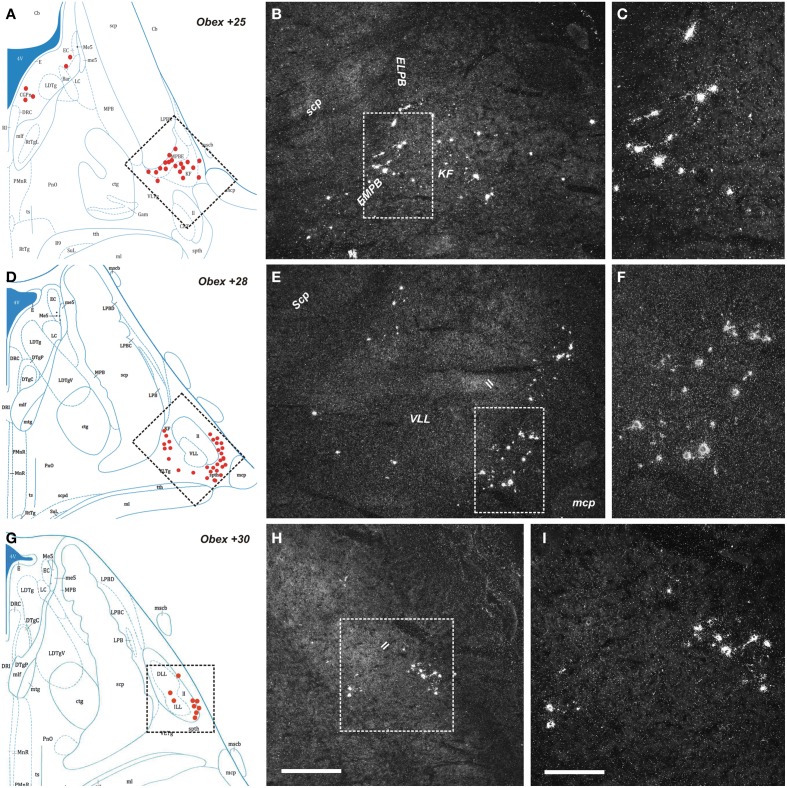
**Distribution of NPS mRNA-positive neurons in human pons. (A,D,G)** Schematic drawings from the atlas by Paxinos et al. (2012), indicating distribution of NPS mRNA-positive cell bodies (red dots) at three different levels (Obex +25, +28, +30). All schematic drawings are presented in higher magnification in the Supplementary Material. Boxed area in **(A,D,G)** show **(B,E,H)**, respectively. **(B,C,E,F,H,I)** Parabrachial cluster harbors numerous NPS mRNA-positive neurons. Note that the NPS neurons are distributed in the external medial/lateral parabrachial nuclei—Kölliker Fuse nucleus—lateral lemniscus region. The boxed area in **(B)**, **(E,H)** show **(C)**, **(F,I)**, respectively. **(A,D,G)** are reproduced from Paxinos et al. (2012) with permission. Scale bars: 500 μm **(H)**, applies to **(B,E,H)**; 200 μm **(I)**, applies to **(C,F,I)**.

The NPS mRNA-expressing neurons are localized in pons, between Obex +20 and Obex +32 mm and distributed in three distinct areas/clusters. (i) The “peri-ventricular cluster”: sparse cells in the central gray matter (CGPn) adjacent to the posterodorsal tegmental nucleus (PDTg) and ventral to the fourth ventricle (Figures 4A–C); (ii) the “peri-coerulear neurons”: very few labeled cells localized dorsally, adjacent to the LC. These cells sometimes intermingle with the LC group, but the pigmented LC noradrenergic neurons were never labeled (Figures 4D–F,H,I); (iii) the “PB cluster”: most of the pontine NPS-expressing neurons belong to this cell group, which starts at Obex +22 mm and ends at around Obex +32 mm. Many labeled cells were found ventral to the superior cerebellar peduncule (scp), in the external part of the medial and lateral PB nuclei (EMPB, ELPB, respectively) and sometimes in the Kölliker-Fuse KF) nucleus (Figures 4D, 5A–C). At Obex +26 mm, the PB cluster split into two sub-clusters surrounding the lateral lemniscus (ll). Thus, one cell group is localized ventral-lateral to the scp but medial to the lateral lemniscus, while the other one is lying on the lateral side of the lateral lemniscus (Figures 5D–F). From approximately Obex +28 mm, some labeled cells were detected in the ventral and intermediate nuclei of the lateral lemniscus (VLL, ILL, respectively; Figures 5G–I).

### Quantitative evaluation of the NPS mRNA-expressing neurons in the human brainstem

**Figure 6:**
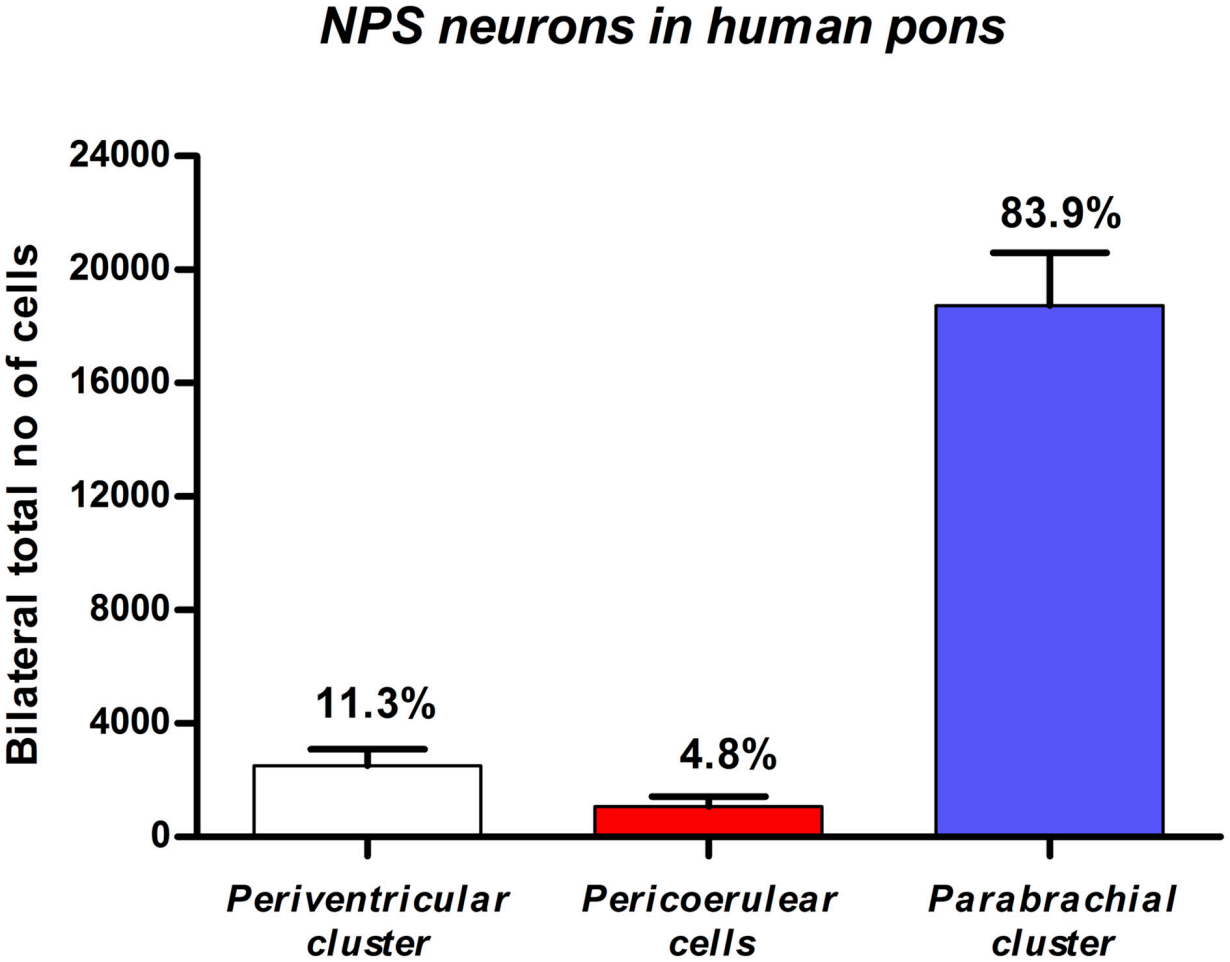
**Quantitative evaluation of NPS mRNA-positive neurons in the human pons**. Stereological methods show that the by far highest number of NPS cells is located in the parabrachial region. Data expressed by mean ±S.E.M; *N* = 3.

Based on stereological methods, the total number of NPS mRNA-expressing cells was estimated to approximately 22 thousands bilaterally (22,317 ± 2411, altogether in both sides). The vast majority (84%) of labeled cells were located in the PB cluster (Figure 6). It is of note that we observed some inter-individual variability in the number of cells (estimated number of NPS mRNA-expressing neurons in the peri-ventricular cluster: 3650, 2150, 1750, cases 1–3, respectively; peri-coerulear cells: 1050, 450, 1700, cases 1–3, respectively; parabrachial cluster: 21,000, 15,050, 20,150, cases 1–3, respectively). Despite this variability, the proportional distribution of NPS cells was apparently different in human subjects, compared to the situation previously described in rodents (Liu et al., 2011). The most prominent difference was that the human peri-coerulear area showed only a few scattered NPS cells in human (ca 5% of all counted neurons), while the peri-coerulear NPS neurons form a prominent cluster in rodents (ca 30% of all counted neurons in mouse; Liu et al., 2011).

### NPS expressing neurons in the rat brainstem

**Figure 7:**
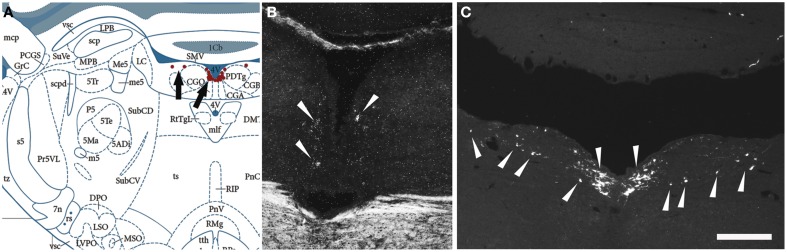
**Comparison of the distribution of NPS mRNA-positive with NPS-immunoreactive neurons in the rat brainstem 1—the periventricular cluster**. **(A)** Schematic drawing from the atlas by Paxinos and Watson (2007), indicating distribution of NPS-positive cell bodies (arrows and red dots) around the fourth ventricle. All schematic drawings are presented in higher magnification in the Supplementary Material. **(B)** shows distribution of the transcript and **(C)** of the peptide immunoreactivity at approximately the same level. Note the similar distribution of the NPS neurons in the periventricular cluster visualized with *in situ* hybridization (NPS mRNA) and immunohistochemistry (NPS peptide). The section in **(C)** is from a colchichine-treated rat. **(A)** is reproduced from Paxinos and Watson (2007), with permission. Scale bar: 200 μm **(C)**, applies to **(B,C)**.

**Figure 8:**
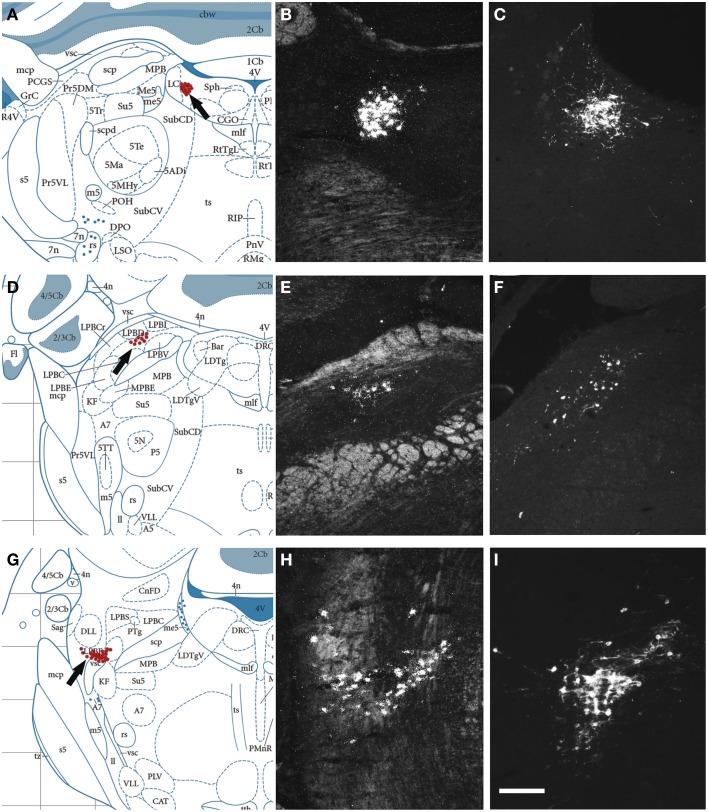
**Comparison of the distribution of NPS mRNA-positive with NPS-immunoreactive neurons in the rat brainstem 2**. **(A,D,G)** Schematic drawings from the atlas by Paxinos and Watson (2007), indicating distribution of NPS-positive cell bodies (arrows and red dots) at three different levels. **(B,E,H)** show distribution of the transcript and **(C,F,I)** of the peptide immunoreactivity at approximately the same levels. All schematic drawings are presented in higher magnification in the Supplementary Material. The present figure shows the following NPS-expressing neuronal groups: (i) the peri-coerulear region **(A–C)**, (ii) the lateral parabrachial nucleus **(D–F)**, and (iii) adjacent to the Kölliker-Fuse nucleus **(G–I)**. Note the similar distribution of the NPS neurons in all three clusters visualized with *in situ* hybridization (NPS mRNA) and immunohistochemistry (NPS peptide). Sections in **(C,F,I)** are from colchichine-treated rats. **(A,D,G)** are reproduced from Paxinos and Watson (2007), with permission. Scale bar: 200 μm **(I)**, applies to **(B**,**C**,**E**,**F**,**H,I)**.

Next, we examined, for a direct comparison, the distribution of NPS expressing neurons in adult Wistar rat both with *in situ* hybridization (transcript) and immunohistochemistry (peptide). In addition to the previously described NPS-expressing cell groups, we found a small cell cluster of NPS neurons medially, adjacent to the posterodorsal tegmental nucleus, just around the midline of the fourth ventricle at Bregma −9.8 to −9.6 mm (the “peri-ventricular cluster”; Figures 7A,B). In agreement with previous studies in rat (Xu et al., 2007), we noted mRNA expression also in (i) a compact cell cluster ventro-lateral to the LC with high numbers of NPS neurons (the “peri-coerulear cluster”; Figures 8A,B); (ii) a cell cluster in the lateral PB nucleus with low numbers of NPS mRNA-positive neurons (the “PB cluster”; Figures 8D,E); and (iii) a cluster lateral to the external part of lateral PB nucleus and dorso-lateral to the KF nucleus with high NPS expression (the “KF cluster”; Figures 8G,H). NPS protein expression in all four cell clusters was confirmed by immunohistochemistry using antibodies to NPS and shown in sections from colchichine-treated rats (Figures 7C, 8C,F,I). Notably, the prominent peri-coerulear NPS cell cluster in rat is virtually missing in human (cf. Figures 8A–C and Figure 4G with Figures 4D–F,H,I).

### NPSR1 mRNA-expressing neurons in the human brainstem

**Figure 9:**
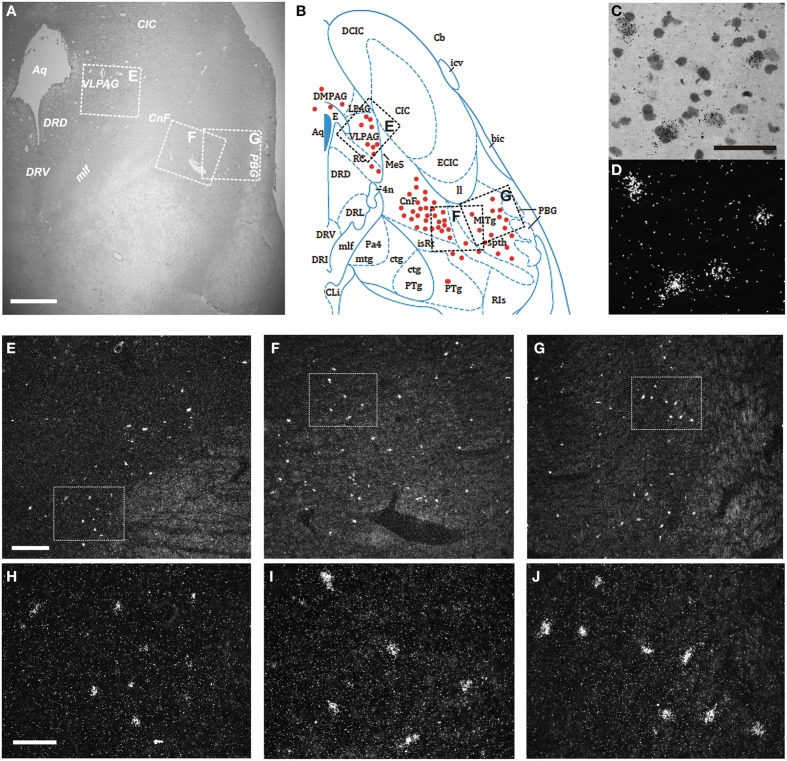
**Distribution of NPSR1 mRNA-positive neurons in the human pons**. **(A,B)** Cresyl violet staining at Obex +35 mm **(A)** and schematic drawing from the same level showing distribution of transcript-positive cells (red dots; **B)**. All schematic drawings are presented in higher magnification in the Supplementary Material. Boxed areas in **(A,B)** indicated with “E,” “F,” and “G” show **(E**,**F,G)**, respectively. **(C,D)** NPSR1 neurons in the cuneiform nucleus in the same, cresyl violet stained section shown in bright- **(C)** and darkfield **(D)**. **(E–J)** Darkfield micrographs showing NPSR1 neurons in the ventrolateral periaqueductal gray **(E,H)** and the cuneiform nucleus—microcellular tegmental nucleus—spinothalamic tract region **(F,G**,**I,J)**. Boxes in **(E,F,G)** show **(H,I,J)**, respectively. **(B)** is reproduced from the atlas of Paxinos et al. (2012), with permission. Scale bars: 1000 μm **(A)**; 50 μm **(C)**, applies to **(C,D)**; 300 μm **(E)**, applies to **(E–G)**; 100 μm **(H)**, applies to **(H–J)**.

Finally, we examined the distribution of the NPS receptor mRNA-expressing neurons in the pons between Obex +15 and Obex +37 mm (Figures 9A,B). Weak labeling of sparse cells was noted in the pontine central gray matter (CGPn), around the LC, ventral to the superior cerebellar penduncle in the medial PB—KF—lateral lemniscus region, and in the caudal part of the laterodorsal tegmental nucleus (LDTg–LDTgV; data not shown). In contrast, strong labeling was found in the rostral LDTg, in the cuneiform nucleus (Figures 9C,D), and medial to the parabigeminal nucleus (PBG) in the spinothalamic tract – microcellular tegmental nucleus (MiTg) region (Figures 9A,B,F,G,I,J). Also, numerous NPSR1 mRNA-expressing cells were noted in all divisions of the periaqueductal gray (dorsomedial, lateral, ventrolateral, raphe cap), especially in the ventrolateral part (VLPAG; Figures 9A,B,E,H). However, there was no NPSR1 mRNA-expression detected in the dorsal or medial raphe nuclei (data not shown).

## Discussion

In the present study we explore the distribution of neurons in the human pons expressing the mRNA of NPS and its receptor. We show that there is a certain agreement but also distinct interspecies differences regarding the distribution of NPS- or NPSR1 mRNA-expressing neurons between the human and rodent brainstem. We focused our studies on the human pons as the NPS-expressing neurons were found in this brainstem region in rat and mouse, the two species, where the anatomy of the NPS system has been studied so far.

### Comparative analysis of the distribution of NPS-expressing neurons in the pons

NPS is a potent neuromodulator in rodents (see for review: Reinscheid and Xu, 2005b), but still the total number of NPS neurons is only about 500 in the mouse brain with an average of 146 and 368 neurons in the peri-coerulear and KF cell clusters, respectively (Liu et al., 2011). In addition, a small NPS-expressing cell group was also described in the lateral PB nucleus, but only in rat (Xu et al., 2007). These data indicate that NPS is one of the least abundant neuropeptides with regard number of neurons in the rodent brain. Similarly, also in the human pons our stereological estimation shows a fairly low number of neurons, that is around 20–25.000 bilaterally, compared to noradrenergic LC neurons (ca. 50,000; Sharma et al., 2010) or hypothalamic orexinergic neurons (50–80,000; Thannickal et al., 2000). Notably, in the first description of the rat NPS-system, low levels of NPS transcript was observed in a few scattered cells in the amygdala and dorsomedial hypothalamic nucleus based on *in situ* hybridization in rats (Xu et al., 2004). However, we could not reproducibly detect these neurons (unpublished observation). Nevertheless, the existence of other NPS-expressing neuronal populations in the human brain, outside the pons, cannot be excluded.

Based on our *in situ* hybridization and stereological studies, the vast majority (84%) of NPS mRNA-expressing cells in the human brainstem are located in the PB region, namely, in the external part of the medial and lateral PB nuclei (EMPB, ELPB), in the KF nucleus and around the adjacent lateral lemniscus. This is in agreement with recent microarray data of the Allen Brain Atlas, showing high NPS mRNA-expression level in human punch samples from the medial/lateral PB-LC area (http://human.brain-map.org/microarray/search/).

The PB-KF complex is a cytoarchitecturally highly organized structure both in human and rodents, even if not all PB subdivisions in rat have corresponding primate homologs (Fulwiler and Saper, 1984; Jia et al., 2005; Paxinos et al., 2009). PB-KF is a relay for visceral afferent information from the brainstem to the forebrain and serves as an integrator of visceral autonomic information and the forebrain regulatory mechanisms of the central autonomic systems (Block and Estes, 1990; de Lacalle and Saper, 2000). It is involved in pneumotaxic, respiratory and cardiovascular control (Block and Estes, 1990) as well as in the modulation of arousal (Fuller et al., 2011). This “PB” NPS cell cluster in human likely corresponds to the “KF cluster” in rat, which is distributed lateral to the external part of lateral PB nucleus and dorso-lateral to the KF nucleus. This neuron cluster is found in both rat and mouse with modest anatomical differences (Liu et al., 2011). In mouse these neurons were found to be glutamatergic, they are innervated by orexinergic fibers, and approximately 50% of them co-express galanin (Liu et al., 2011). Further studies are required to determine the neurochemical profile of NPS neurons in human.

Comparative anatomical studies have shown that the connections of the EMPB and ELPB nuclei are highly conserved and exhibit a large homology between human and rat (de Lacalle and Saper, 2000). Namely, the LPBE nucleus is a relay nucleus of visceral information from the nucleus tractus solitarii to forebrain limbic structures, e.g., the central nucleus of amygdala and the bed nucleus of the stria terminalis (BNST). The EMPB nucleus provides topographic projections to the contralateral ventroposterior parvicellular nucleus of the thalamus (VPpc), which projects to the insular cortex. Thus, the EMPB relays the entire spectrum of visceral sensation to conscious appreciation (de Lacalle and Saper, 2000). Recently, the pivotal role of glutamatergic projections from the ELPB nucleus, as a relay for arousal signal to the forebrain in obstructive sleep apnea syndrome (OSAS), was described (Chamberlin, 2013; Yokota et al., 2015). Despite this, no significant association of NPS gene polymorphisms with OSAS or OSAS variables could be demonstrated (Sanchez-De-La-Torre et al., 2011). However, and interestingly, the NPSR1 gene was originally described as an asthma susceptibility gene (Laitinen et al., 2004), and significant SNP and haplotype associations of NPSR1 gene with asthma was established in several independent populations (Pietras et al., 2011).

The KF nucleus is a fundamental component of the central respiratory circuitry, postnatally coordinating the pulmonary motor responses to the blood oscillations of pO2, pCO2, and pH. But it also has a pivotal role prenatally by inhibiting the respiratory reflex (Lavezzi et al., 2004). In victims of sudden infant death syndrome the KF nucleus shows clear signs of developmental immaturity and low levels of BDNF immunostaining (Lavezzi et al., 2014). Ohm and Braak earlier showed that the gray matter around the ventral edge of the superior cerebellar pedunculus is especially vulnerable in Alzheimer's pathology: it exhibits severe neuronal loss and abundant tau pathology (Ohm and Braak, 1988). Further systematical analysis is required to determine the vulnerability of the NPS neurons in the PB region in neurodegenerative conditions.

Surprisingly, the NPS-expressing cell cluster adjacent to the LC, which is prominent in the rat and mouse (Xu et al., 2007; Liu et al., 2011), is virtually missing in human, only representing a few scattered neurons (4–5% of all NPS cells). In rodents, NPS neurons in the peri-coerulear cluster are glutamatergic, they are surrounded by a dense network of orexinergic and galaninergic fibers (Liu et al., 2011), they are stress-reactive (Liu et al., 2011; Jüngling et al., 2012) and involved in the regulation of arousal (our unpublished data). The relevance and reason of the apparent lack of a prominent peri-coerulear NPS neuron cluster in human, in contrast to mice and rats, is currently unknown. To examine the distribution of NPS mRNA-expressing neurons in diurnal rodents (e.g., degu, Lee, 2004) or in nocturnal vs. diurnal non-human primates (e.g., owl monkey (O'Keefe et al., 1998) vs. rhesus monkey) may be a subject of further studies. Importantly, we were able to perform systematic neuropathologic evaluation of the cases involved in our study (see Table 1). In cases 1 and 3 the rostral and caudal part of the brainstem did not show any signs of neurodegenerative or vascular pathology. It must, however, be noted that in case 2 we observed an incidental cortico-subcortical tauopathy in an individual without any neurological symptoms. In this case we did see scattered tau positive neurons in the substantia nigra but not in the periaqueductal and ventral tegmentum region of the mesencephalon or lower pons or medulla oblongata. Interestingly, this case showed the lowest NPS cell counts in the peri-coerulear (and also in the PB) region. It must be emphasized, however, that the proportional distribution of NPS neurons in the peri-coerulear region was similarly low in all of the examined human cases, compared with rodents (Liu et al., 2011).

Finally, we found a smaller cluster of human NPS mRNA-expressing neurons in the pontine central gray matter (CGPn) adjacent to the posterodorsal tegmental nucleus (PDTg) at around Obex +21 to +23 mm. We termed it as “peri-ventricular cluster.” It constitutes around 11% of all NPS cells in the human pons. The PDTg nucleus contains AChE immunopositive neurons (Huang et al., 1992), and the surrounding neuropil includes a dense network of substance P immunoreactive fibers. Importantly, we found this NPS cell cluster also in rat at around Bregma −9.8 to −9.6 mm, both by *in situ* hybridization (transcript) and immunostaining after colchichine-treat (peptide). Thus, here we describe a novel cell cluster of NPS-expressing neurons in rat, which exists also in the human brain. The neurochemical and functional characterization of these neurons require further studies.

### NPSR1 mRNA-expressing neurons in the human pons

Next, we examined the distribution of NPSR1 mRNA in the human pons. A few weakly labeled neurons were found in those regions, where the NPS peptide mRNA-expressing neurons localize (pontine central gray matter, PB—lateral lemniscus region). The majority of NPSR1 mRNA-expressing cells in the pons, however, distribute (i) in the periaqueductal gray and (ii) in the rostral pedunculopontine nucleus—cuneiform nucleus—microcellular tegmental nucleus region.

Similarly to the PB region, the periaqueductal gray (PAG) is also a cytoarchitecturally and neurochemically highly complex intergrative area, both in human and rodent brain (Behbehani, 1995; Fu et al., 2010). It is involved in the emotional regulation (e.g., in circuitries underlying fear, depression and anxiety), but also in autonomic control and pain (Behbehani, 1995; Satpute et al., 2013). Its ventrolateral subdivision (VLPAG), where the NPSR1 mRNA-expressing neurons are especially abundant, is critical for the expression of passive coping responses to non-immediate “distal” danger (Johnson et al., 2004), but neurons in the vlPAG contribute to the regulation of REM sleep as well (Luppi et al., 2012). The NPSR1 mRNA-expression in the PAG is detected also in rodents (Xu et al., 2007; Clark et al., 2011). In fact, the anxiolytic effect of NPS is well established in different animal models (Xu et al., 2004; Jüngling et al., 2008; Pulga et al., 2012; Wegener et al., 2012).

The pedunculopontine tegmental nucleus (PPTg) contains primary cholinergic but also GABAeric and glutamatergic neurons. Its functions are multiple, including the regulation of REM sleep, stimulus-reward learning and visual orientation (Wang and Morales, 2009). In addition, the PPTg nucleus and cuneiform nucleus are both important components of the mesencephalic locomotor region, facilitating muscle tone during the initiation of locomotion (Alam et al., 2011). This region is a promising target of deep brain stimulation in Parkinson's disease (Alam et al., 2011; Xiang et al., 2013).

Interestingly, in the rat, the NPSR1 mRNA-expression is high in the oral part of the pontine reticular nucleus and in the median raphe (Xu et al., 2007; Clark et al., 2011), but we did not find labeled cells in these regions in the human, suggesting interspecies differences also in the distribution of NPSR1 between human and rat.

## Conclusion

In the present study we show that the vast majority (84%) of NPS mRNA-expressing neurons in the human pons is distributed in the PB—lateral lemniscus region. The total number of NPS mRNA-expressing neurons in the human pons is around 20–25.000 bilaterally, which supports the notion that NPS is, just like in rodents, a rare neuropeptide also in the human brain with regard to the number of expressing neurons. In addition, here we describe a smaller cell cluster of NPS expressing neurons in the pontine central gray matter both in human and rat, which has not been identified previously even in rodents. In human, this latter cluster contains approximately the 11% of all pontine NPS cells. Both NPS and NPSR1 in the human pons are preferentially localized in integrative relay regions (PB complex in case of NPS, and periaqueductal gray in case of NPSR1), which represent interfaces between visceral and forebrain autonomic centers for higher order processes, such as emotional behavior. Also, the PB/KF complex, where most of the NPS neurons are localized, was recently recognized as a key brain area in the pathophysiology of obstructive sleep apnea and sudden infant death syndrome (Lavezzi et al., 2014; Yokota et al., 2015). The distribution of NPS- and NPSR1 mRNA-expressing neurons exhibits just partial agreement between rodents and human. Perhaps the most remarkable interspecies difference is that the NPS-expressing cell cluster adjacent to the LC, which is prominent in the rat and mouse, is virtually missing in human or represented only by a few scattered neurons. This and the other reported differences must be considered in the research for new pharmacotherapeutical interventions.

### Conflict of interest statement

The authors declare that the research was conducted in the absence of any commercial or financial relationships that could be construed as a potential conflict of interest.

## Abbreviations

4V: fourth ventricle
Aq: aqueduct
BNST: bed nucleus of the stria terminalis
Cer: cerebellum
CGPn: pontine central gray
CIC: central colliculus inferior
CnF: cuneiform nucleus
DR: dorsal raphe
DRD: dorsal raphe dorsal subdivision
DRV: dorsal raphe ventral subdivision
ILL: intermediate nucleus of the lateral lemniscus
KF: Kölliker-Fuse nucleus
LC: locus coeruleus
ll: lateral lemniscus
LPBE: external part of the lateral parabrachial nucleus
mcp: middle cerebellar peduncule
MiTg: microcellular tegmental nucleus
ml: medial lemniscus
mlf: medial longitudinal fasciculus
MPBE: external part of the medial parabrachial nucleus
PAG: periaqueductal gray matter
PB: parabrachial
PBG: prabigeminal nucleus
PDTg: posterodorsal tegmental nucleus
PPTg: pedunculopontine tegmental nucleus
scp: superior cerebellar peduncule
VLL: ventral nucleus of the lateral lemniscus
VLPAG: ventrolateral periaqueductal gray
VPpc: ventroposterior parvicellular nucleus of the thalamus.
