# Supplementary material for: Neuropeptide S- and Neuropeptide S receptor-expressing neuron populations in the human pons
Source: Front Neuroanat. 2015 Sep 25;9:126. doi: 10.3389/fnana.2015.00126 (PMC4585187; doi:10.3389/fnana.2015.00126)
Supplement: Supplementary file 1 [file Presentation1.PDF]

NEUROPEPTIDE S- AND NEUROPEPTIDE S RECEPTOR-EXPRESSING NEURON POPULATIONS IN THE HUMAN PONS

ADORI C ET AL, 2015

SUPPLEMENTARY MATERIAL

Content:

Supplementary figures 1-10

Legends for Supplementary figures

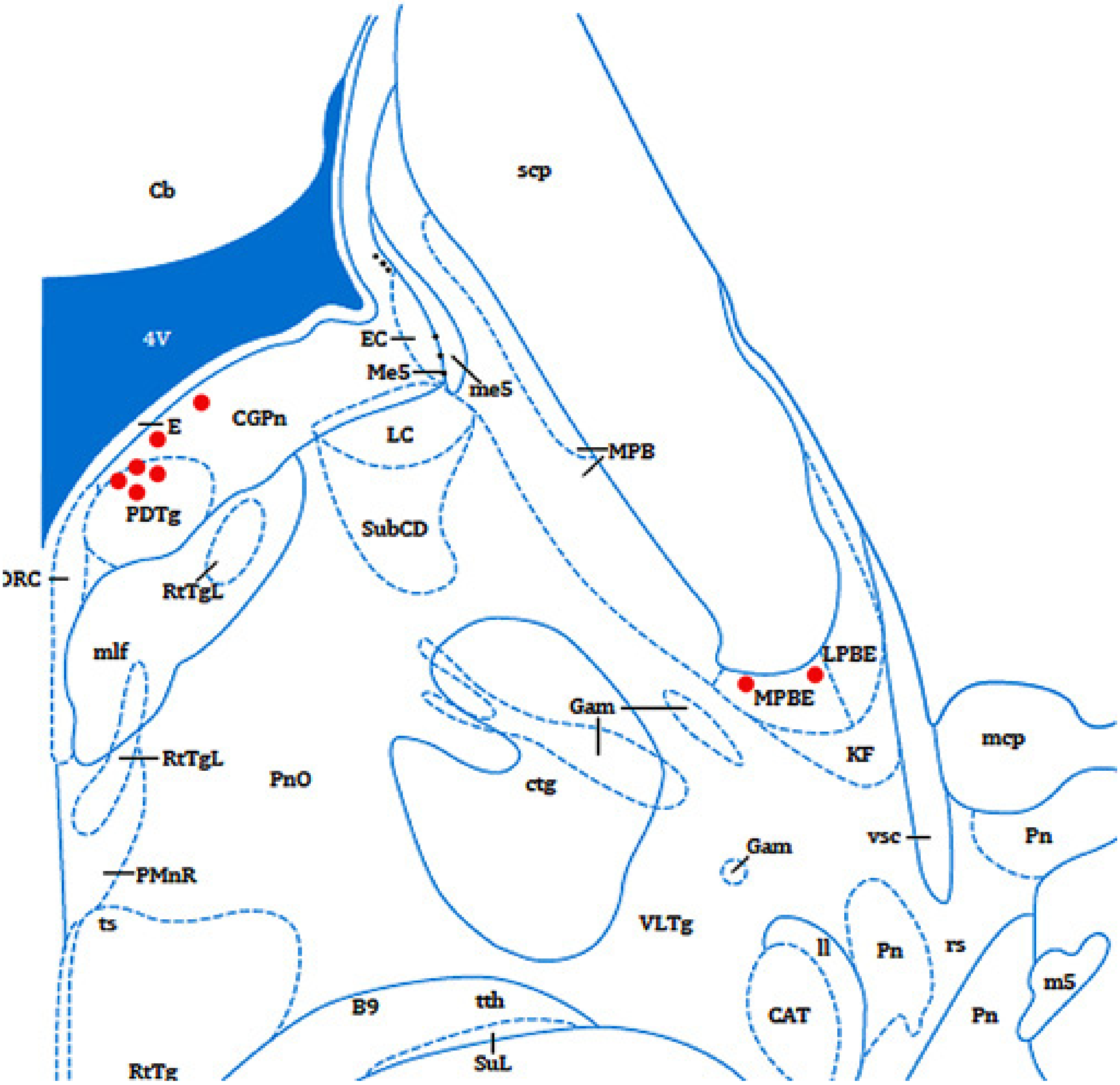

Supplementary figure 1

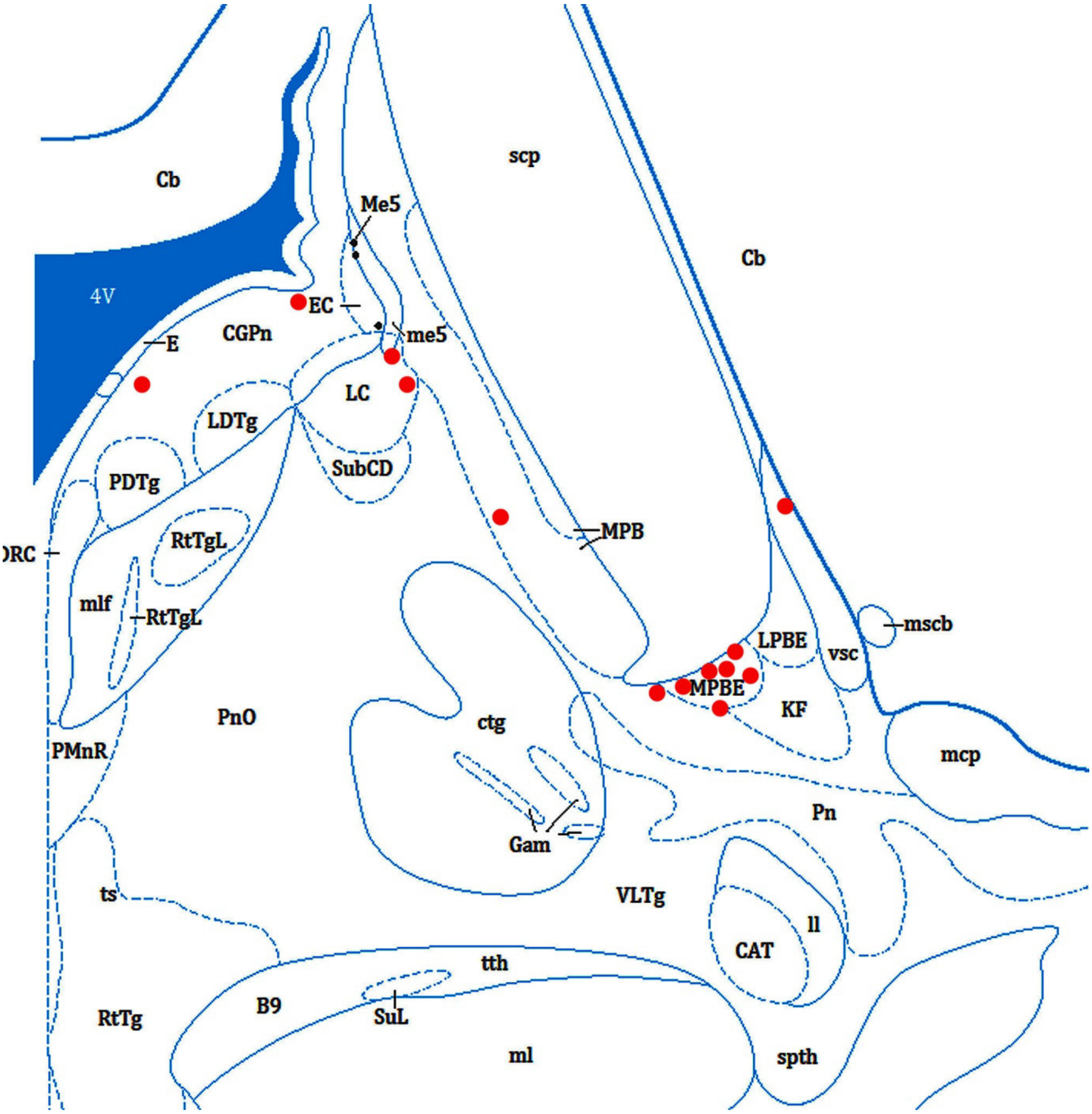

Supplementary figure 2

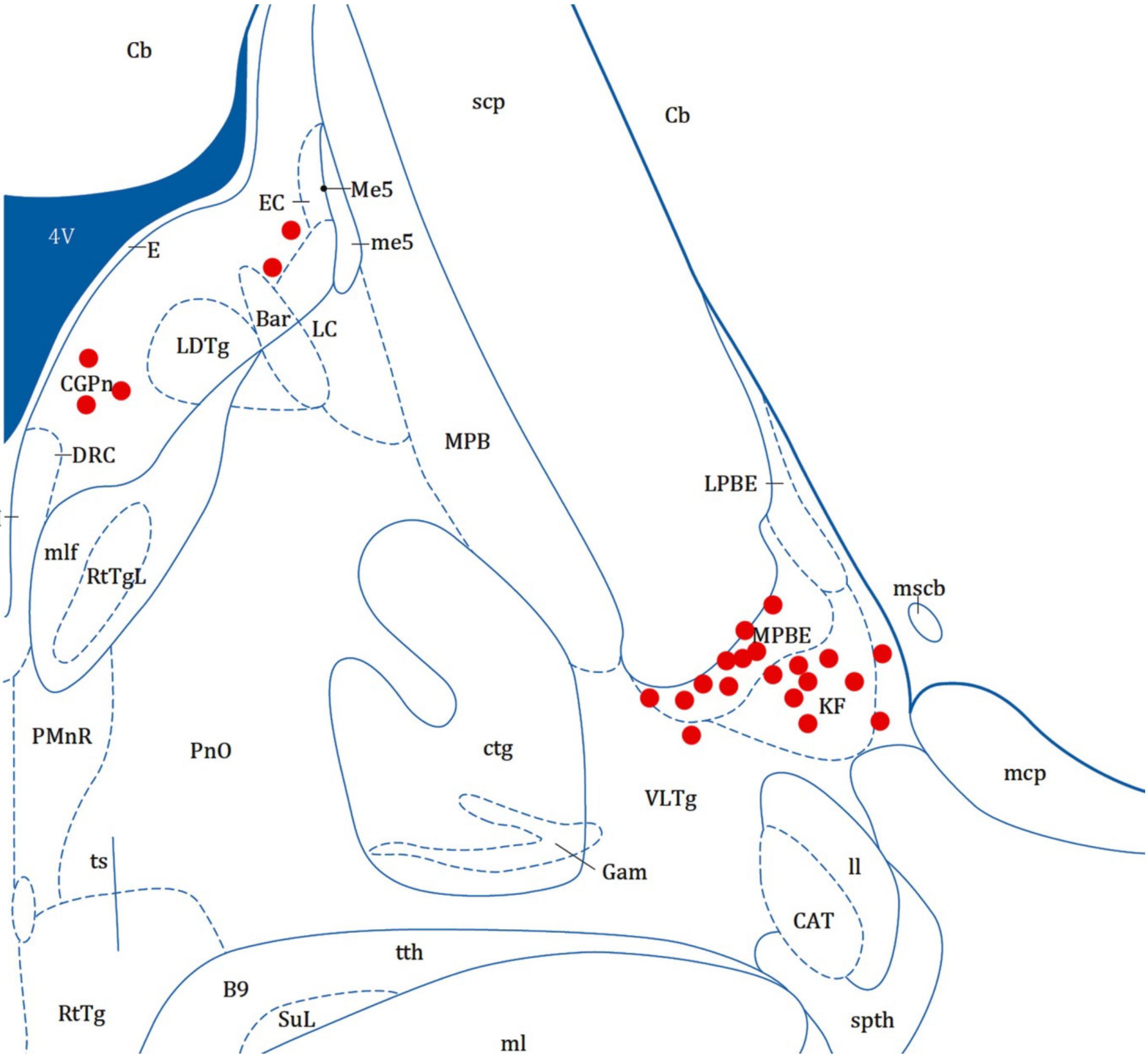

Supplementary figure 3

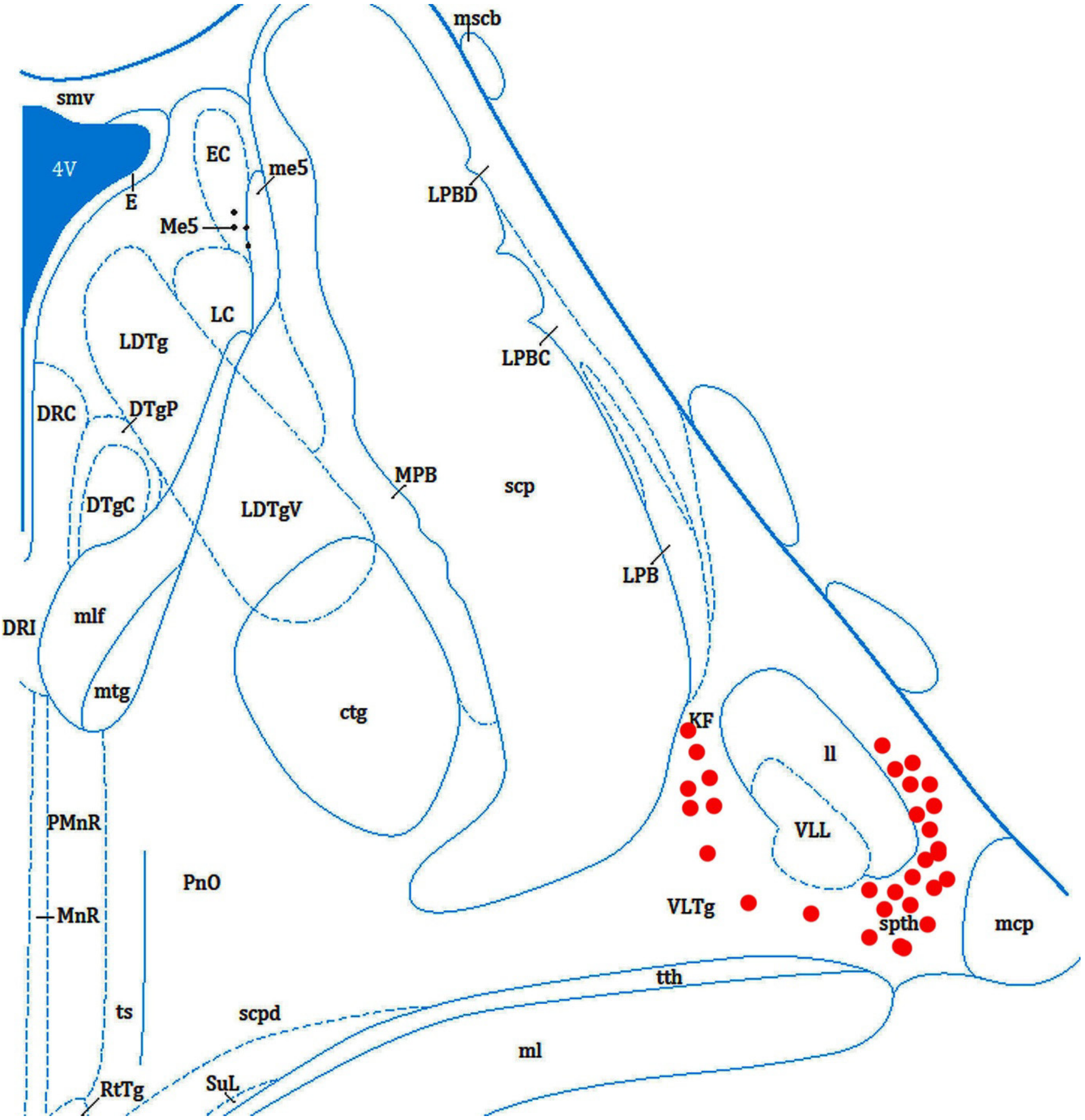

Supplementary figure 4

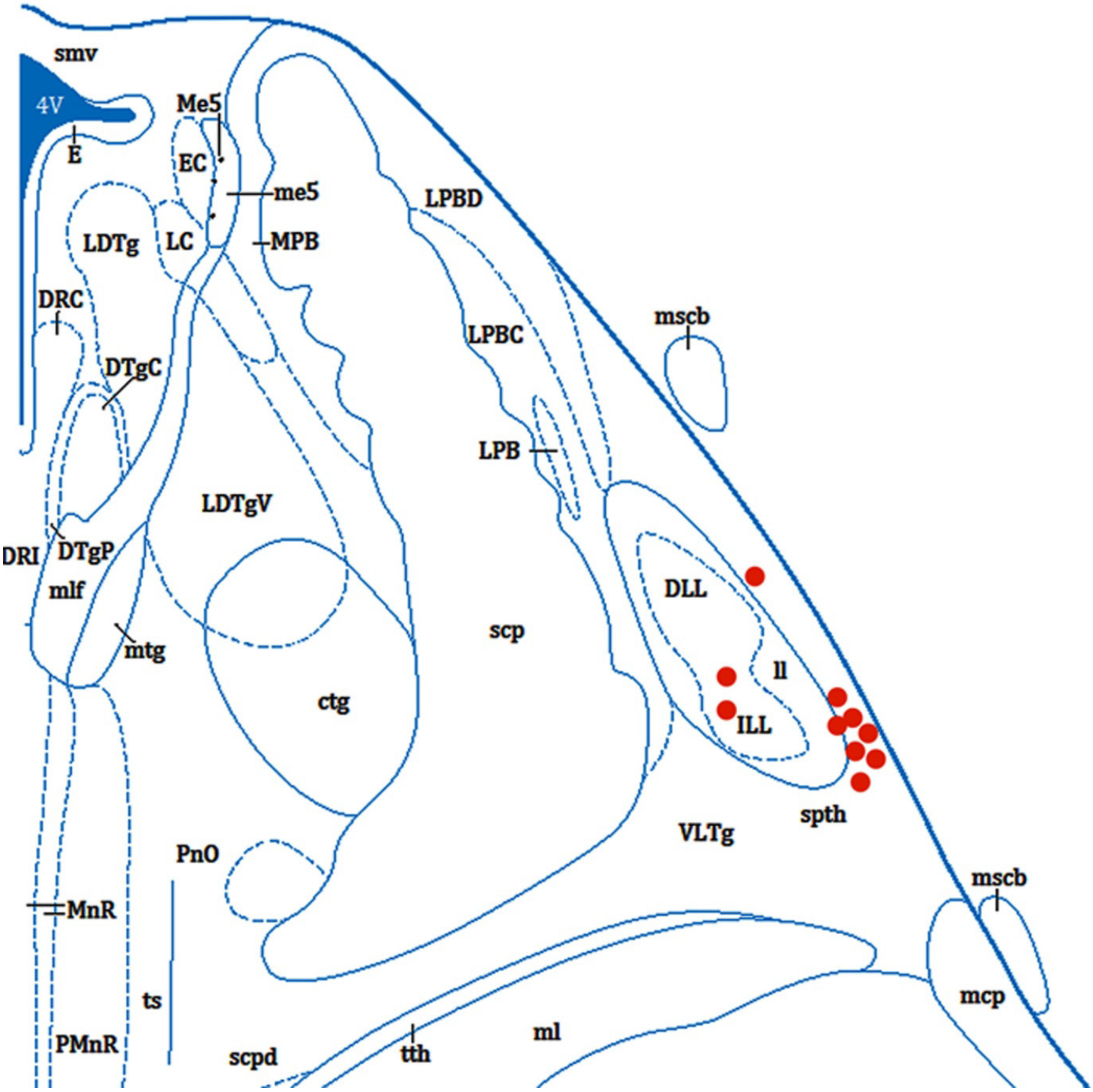

Supplementary figure 5

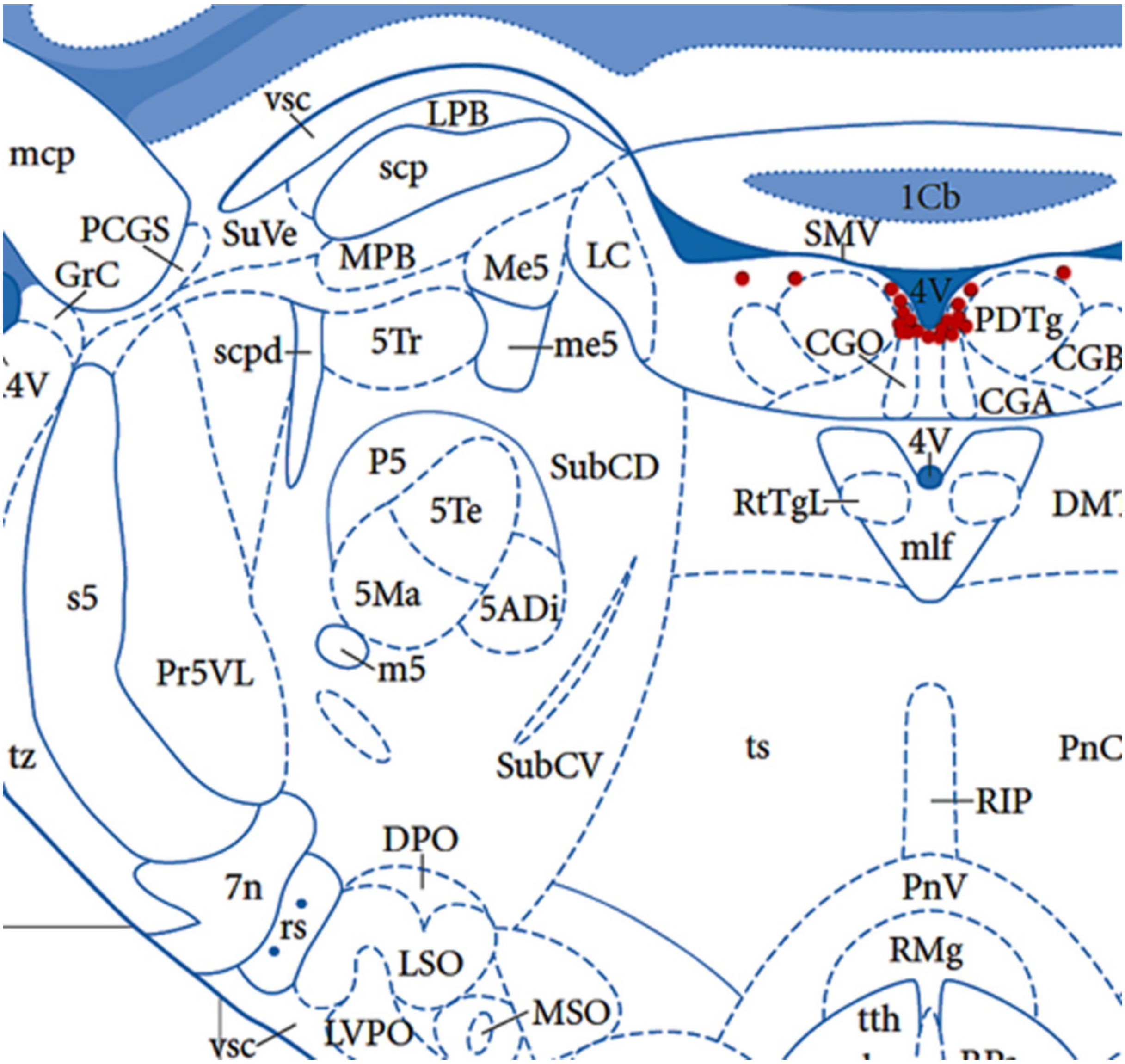

Supplementary figure 6

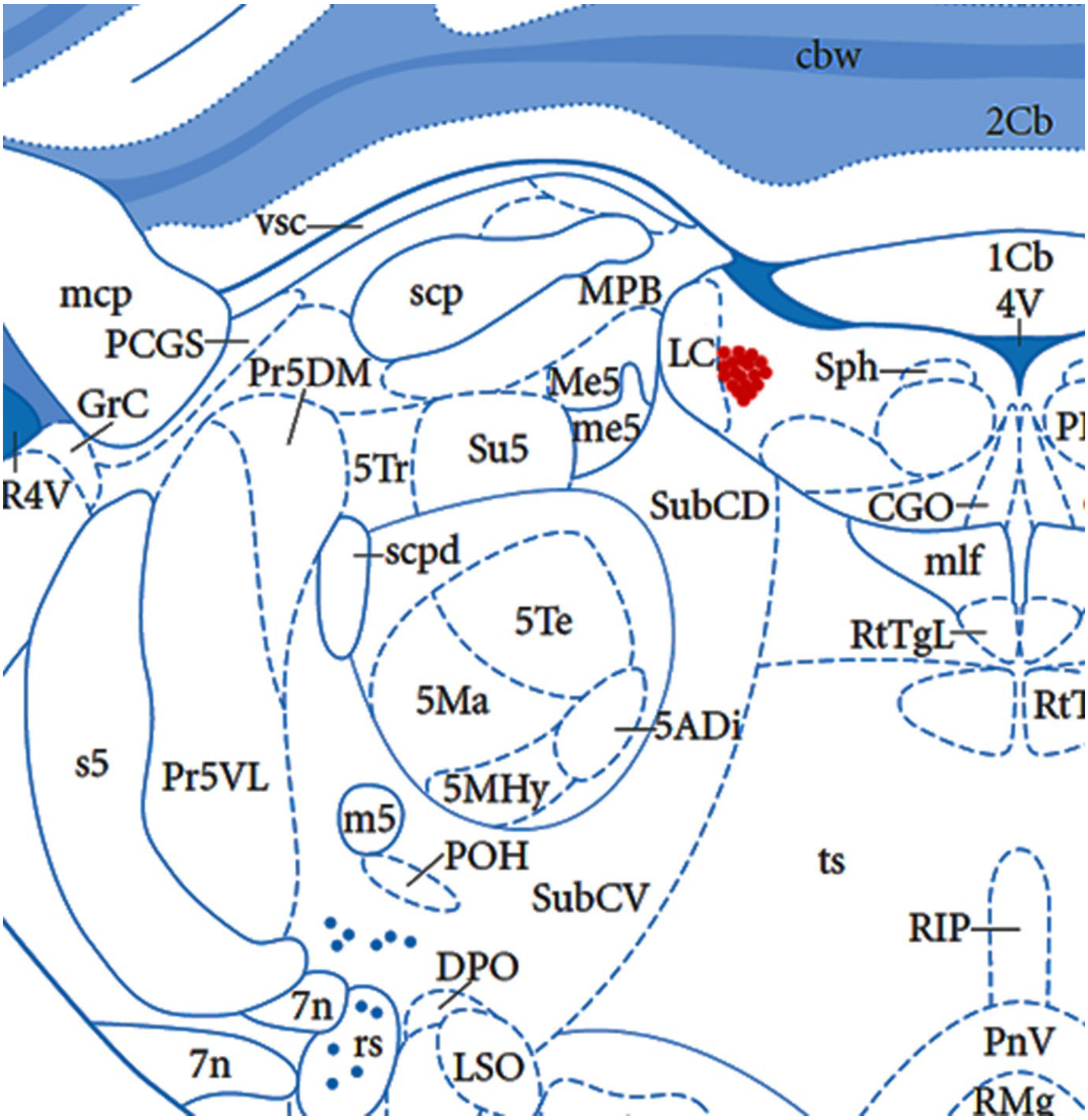

Supplementary figure 7

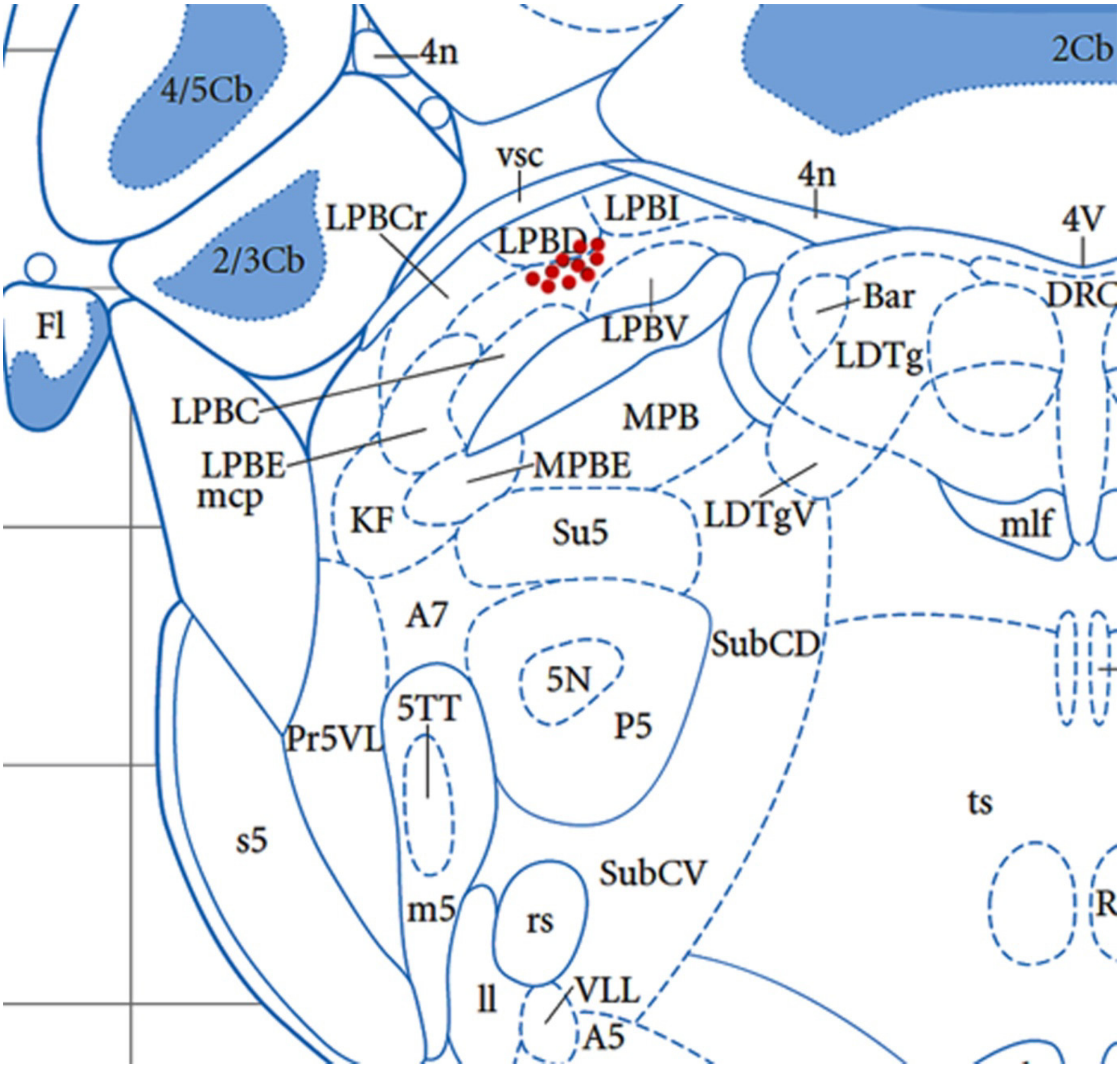

Supplementary figure 8

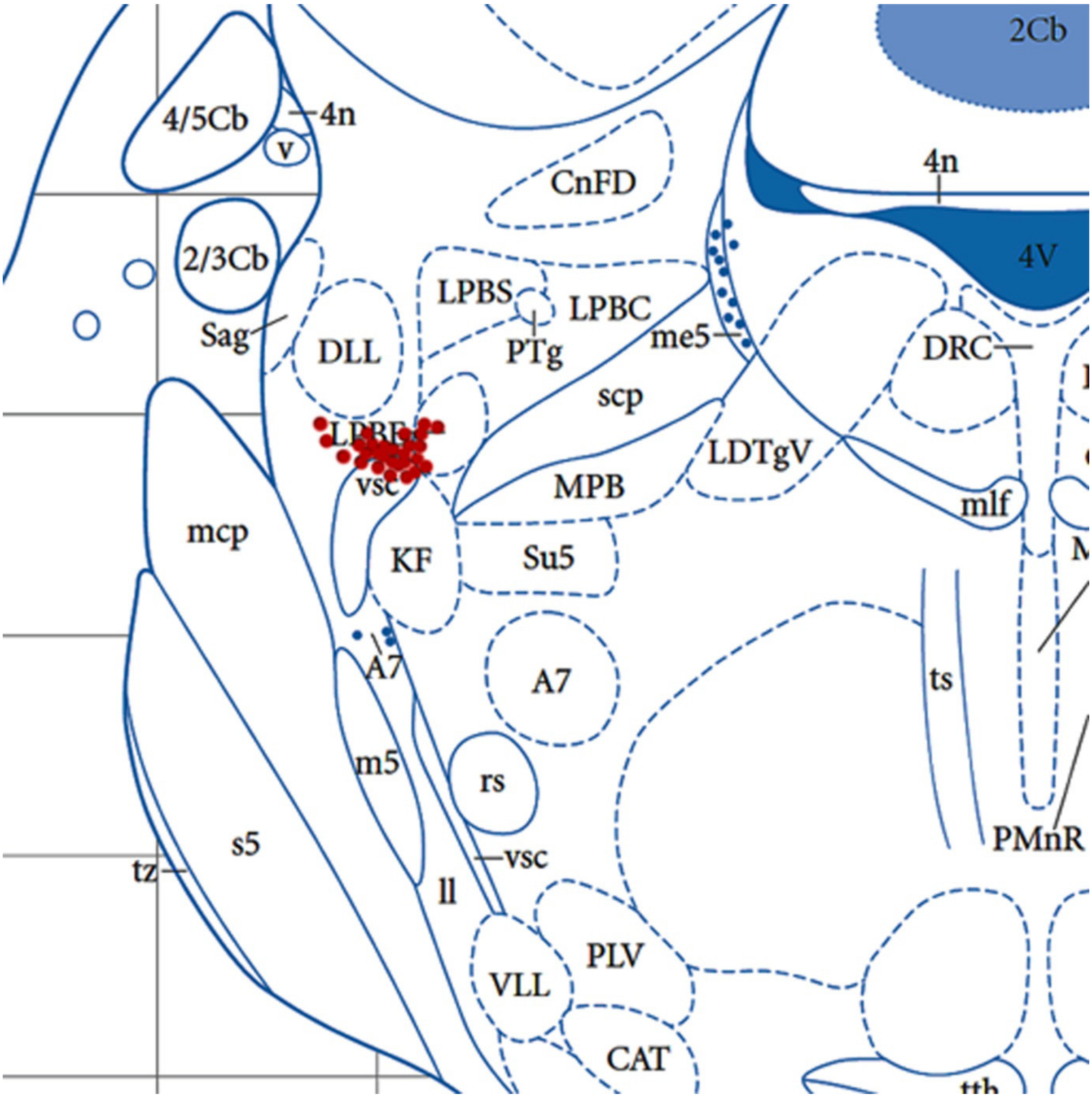

Supplementary figure 9

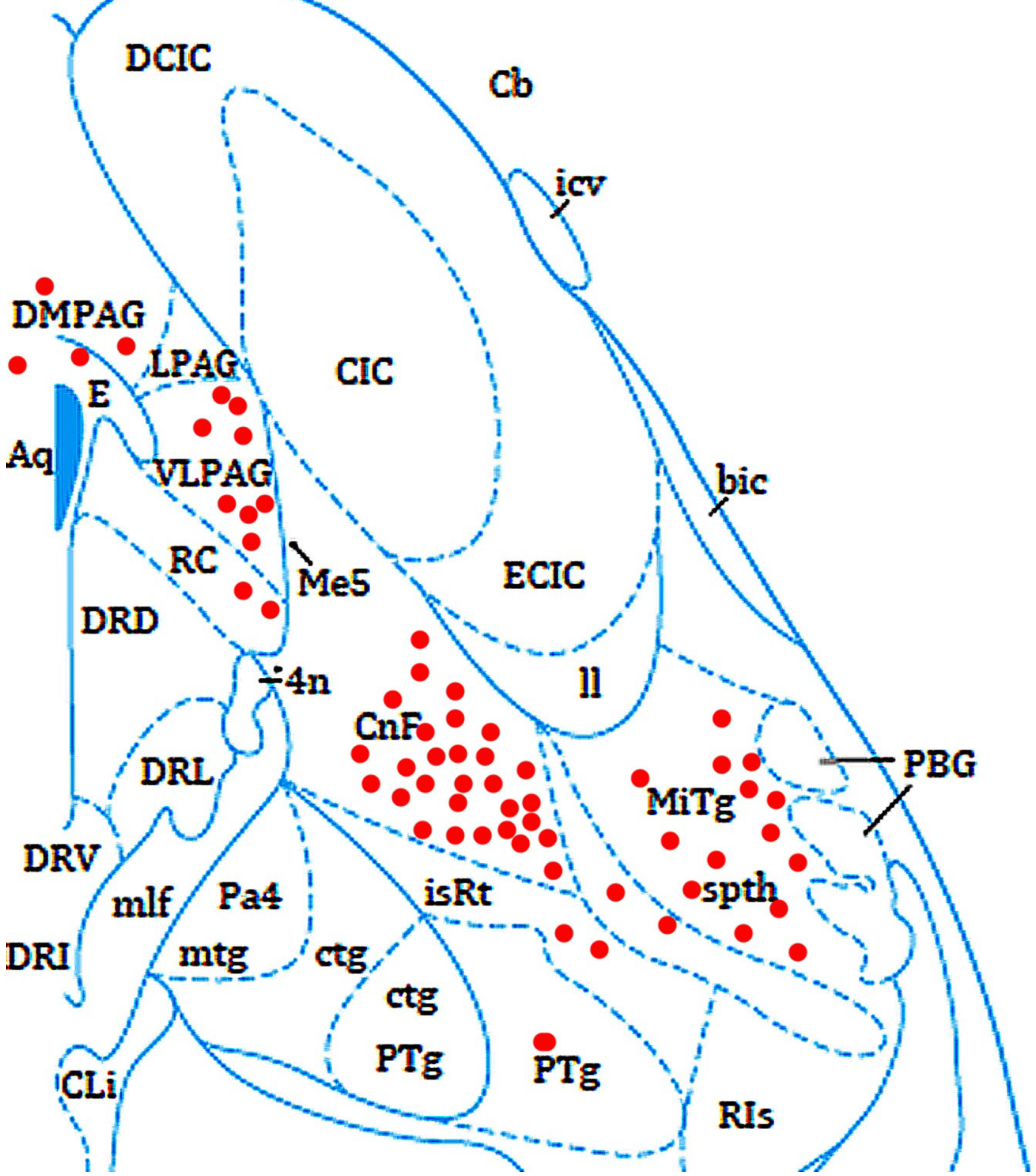

Supplementary figure 10

SUPPLEMENTARY FIGURE LEGEND

All supplementary figures are reproduced from (Paxinos et al., 2012) or (Paxinos and Watson, 2007) with permission.

Supplementary figures 1-2 (for Figure 4) Schematic drawings from the atlas by (Paxinos et al., 2012), indicating distribution of NPS mRNA-positive cell bodies (red dots) at two different levels (Obex +23 and +24, Supplementary figures 1, 2, respectively).

Supplementary figures 3-5 (for Figure 5) Schematic drawings from the atlas by (Paxinos et al., 2012), indicating distribution of NPS mRNA-positive cell bodies (red dots) at three different levels (Obex +25, +28 and +30, Supplementary figures 3, 4, 5, respectively).

Supplementary figure 6 (for Figure 7) Schematic drawing from the atlas by (Paxinos and Watson, 2007), indicating distribution of NPS-positive cell bodies (red dots) around the fourth ventricle.

Supplementary figure 7-9 (for Figure 8) Schematic drawings from the atlas by (Paxinos and Watson, 2007), indicating distribution of NPS-positive cell bodies (red dots) at three different levels (pericoerulear, parabrachial and Kölliker-Fuse clusters of NPS-expressing neurons, Supplementary figures 7, 8, 9, respectively).

Supplementary figure 10 (for Figure 9) Schematic drawing from the atlas by (Paxinos et al., 2012), indicating distribution of NPSR1 mRNA-positive cell bodies (red dots) at Obex +35.
